# A task-independent neural representation of subjective certainty in visual perception

**DOI:** 10.3389/fnhum.2015.00551

**Published:** 2015-10-09

**Authors:** Johannes Heereman, Henrik Walter, Hauke R. Heekeren

**Affiliations:** ^1^Biological Psychology and Cognitive Neuroscience, Department of Education and Psychology, Freie Universitaet BerlinBerlin, Germany; ^2^Berlin School of Mind and Brain, Humboldt Graduate School, Humboldt-Universitaet zu BerlinBerlin, Germany; ^3^Division of Mind and Brain Research, Department of Psychiatry and Psychotherapy, Campus Mitte, Charité Universitaetsmedizin BerlinBerlin, Germany

**Keywords:** decision making, confidence, subjective certainty, perception, fMRI

## Abstract

Am I really sure? This is a question not only scientists ask themselves but practically everybody every day. A recent study provides behavioral evidence supporting the view that one’s subjective confidence in a decision (i.e., feeling sure that a decision is correct) is represented in a task-independent format. Previous neuroimaging studies identified neural correlates of decision confidence but whether or not these are task-dependent remains unclear. Here, combining two perceptual decision tasks with functional magnetic resonance imaging (fMRI), we provide neural evidence for a task-independent representation of degrees of subjective certainty (i.e., a neural representation of subjective certainty that remains constant across two visual tasks). Importantly, due to the constant stimulus-intensity used this result is independent of task-difficulty and stimulus properties. Our data provide strong evidence for a generic mechanism underlying the computation of subjective perceptual certainty in vision.

## Introduction

Am I really sure? This is a question not only scientists ask themselves but practically everybody everyday. Shall I get a less interesting but better paying job? Shall I finally end my annoying relationship? Or give it another try? These are decisions we make with more or less subjective certainty. Generally, when we make decisions, we do that with varying degrees of subjective certainty, or confidence. The mechanisms of the emergence of degrees of subjective certainty have been investigated in humans for more than a century (e.g., Peirce and Jastrow, 1884; Vickers, 1979; Fleming et al., 2010, 2012; Pleskac and Busemeyer, 2010; Hebart et al., 2014; Zizlsperger et al., 2014; Gherman and Philiastides, 2015) and more recently in animals (Kepecs et al., 2008; Kiani and Shadlen, 2009; Komura et al., 2013).

So far research has mostly focused on process models of certainty (e.g., Peirce and Jastrow, 1884; Vickers, 1979; Pleskac and Busemeyer, 2010; Hebart et al., 2014; Gherman and Philiastides, 2015) rather than representational models. While process models describe system dynamics and computational mechanisms, representation models describe the processing device itself and the way an entity is represented (such as by a feature list).

An outstanding question on the representational level is how degrees of subjective certainty are represented on the neural level. For example it may be the case that in two different tasks distinct brain areas represent subjective certainty. In line with this view, Kiani and Shadlen (2009) report that choice certainty is computed along with the decision in sensory-motor neurons in Area LIP, i.e., represented in a task-specific fashion. On the other hand, De Gardelle and Mamassian (2014) provide behavioral evidence supporting the view that one’s subjective confidence in a decision is represented in a task-independent format. In this study subjects either performed two identical or two different perceptual task-trials in succession. After each pair of trials they had to indicate, in which of the two trials they were more confident in the correctness of their decision. The authors reason, that if confidence was task specific, then comparing confidence across two different tasks should be harder than comparing confidence across two instances of the same task. They found no difference between the two conditions, supporting the view that confidence is accessed as an abstract and task-independent quantity. In consequence, as an alternative hypothesis to the task-specific representation of subjective certainty, subjective certainty in different tasks could share a common neural substrate. Previous neuroimaging studies identified neural correlates of decision confidence, i.e., the degree of belief subjects have in the correctness of their choice (e.g., Fleming et al., 2012; Hebart et al., 2014). The brain area most consistently found to code for post decisional confidence in humans is the dorsomedial prefrontal cortex (DMPFC; Fleck et al., 2006; Fleming et al., 2012; Hebart et al., 2014). Other areas reported to carry a confidence signal in humans include the dorsolateral prefrontal cortex (DLPFC; Fleck et al., 2006), the ventral striatum and the right anterior insula (Hebart et al., 2014) as well as right posterior parietal cortex and bilateral middle frontal gyrus (MFG; Fleming et al., 2012). Crucially, the dependence of these neural representations on a particular task remains unclear. Previous studies couldn’t address this question because they either only looked at one task at a time (e.g., Fleming et al., 2012; Hebart et al., 2014), didn’t control for difficulty (Fleck et al., 2006), or were pure behavioral studies (e.g., De Gardelle and Mamassian, 2014).

The hypothesis we investigate here is, whether there is a task-independent neural representation of subjective certainty. We reasoned that in a brain region, to be considered the neural substrate of a task-independent certainty-representation, BOLD signal should vary with the degree of subjective certainty, independent of the kind of task. For this study we therefore developed a functional magnetic resonance imaging (fMRI)-design combining two tasks, a color and a motion detection task.

Because most researchers in the field are interested in the relationship between performance and confidence (belief in the correctness of a choice; for critical treatments of this approach see Gigerenzer et al., 1991; Drugowitsch et al., 2014) the usual approach is to calibrate stimulus intensities to predefined levels of performance. In the experimental trials subjects indicate their (binary) decision first and only then rate their confidence (e.g., Fleming et al., 2010; Pleskac and Busemeyer, 2010; Fleming et al., 2012). Crucially, Baranski and Petrusic (2001) report that in this design confidence processing occurs both parallel to choice and after choice. In addition, Pleskac and Busemeyer (2010) and Yu et al. (2015) show that in this design subjects continue to accumulate evidence after choice, so that the confidence ratings are not based on the same evidence as the decision. Finally, Boldt and Yeung (2015) report evidence supporting the view that shared mechanisms underlie post decisional confidence ratings and post decisional error detection. Therefore, because we were interested in the degree of certainty leading to choice (and neither in post decisional confidence nor in error detection) and it’s neural correlates we flipped the usual response order and asked for certainty ratings first and let subjects only then indicate their decision. Also, because the quantity of interest here is subjective certainty, in contrast to previous studies, we calibrated stimulus intensity to a predefined average level of subjective certainty instead of performance.

## Materials and Methods

### Participants

A total of 24 healthy right-handed volunteers with normal or corrected to normal vision participated in the experiment. Eligibility was assessed with a general health questionnaire and an fMRI safety screening form. None had a history of psychiatric or neurological disorder. Two subjects aborted the experiment due to dizziness. Two additional subjects were excluded due to excessive head motion. The latter 2 subjects’ data were used for behavioral analyses, resulting in a sample of 22 subjects (mean age = 23.5, min = 20, max = 28, 13 female). (The final fMRI analyses were carried out with data obtained from the remaining 20 subjects (mean age = 23.3, min = 20, max = 28, 11 female). The study was approved by the local ethics committee at the Freie Universitaet Berlin, Germany, and carried out in accordance to the Declaration of Helsinki. All subjects gave informed written consent before the study.

### Stimuli

**FIGURE 1:**
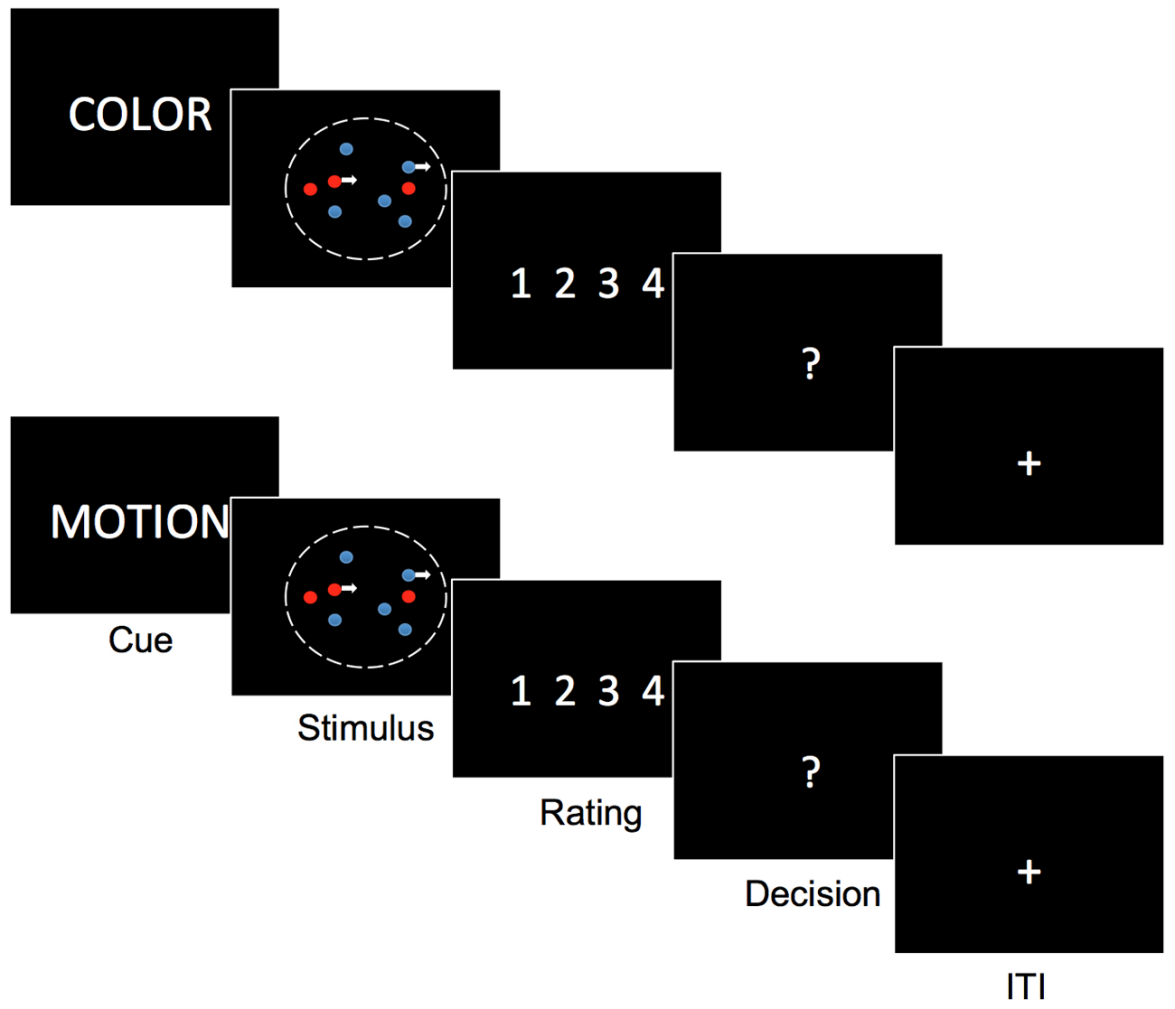
**Task description**. subjects see on the display a cloud of colored moving dots. In the beginning of each trial they are cued whether to attend motion and ignore color or attend color and ignore motion. Depending on the cue after stimulus presentation (750 ms) they have to rate their degree of certainty that motion was to the left or right or that one color was more present than the other (blue or red). Only after the certainty rating, they indicate the respective direction or color. Task modified from Kayser et al. (2010).

Participants performed a color-motion-detection-task (see **Figure 1**). The dots were blue and red, presented on a black background and were drawn in a circular aperture for the duration of one video frame (60 Hz). The dots were redrawn after ∼50 ms at either a random location or a neighboring spatial location to induce apparent motion. The resulting motion effect appeared to move between 3 and 7°/s, and the dots were drawn at a density of 16.7 dots per degree/second. For the color manipulation we used the values from Kayser et al. (2010). The two values were red: RGB = (255 65 2) and blue: RGB = (5 137 255). A subportion of the dots was assigned the target color while the rest of the dots was evenly divided between blue and red. The subset of dots representing the coherent feature (motion and/or color) was changed from frame to frame so that the subset of coherent dots on one frame was not the same as the subset of coherent dots on the previous frame. The task was implemented using Psychtoolbox-3 (Brainard, 1997; Pelli, 1997; Kleiner et al., 2007). In order to avoid floor and ceiling effects, previous to testing stimulus intensities (coherence values) were calibrated to an average level (2.5 on a scale of 1–4) of subjective certainty in a subject-specific manner. The resulting average coherence-levels were 0.178 (min = 0.14, max = 0.217) for motion and 0.197 (min = 0.137, max = 0.269) for color. These subject-specific stimulus intensities were then held constant throughout the whole experiment. Subjects indicated their responses using a four-button-response-box in the right hand (for ratings) and a two-button-response-box in the left hand (for direction and color indication). (Current Designs, Philadelphia).

#### Task

Subjects saw a cloud of colored moving dots. In the beginning of each trial a verbal cue appeared on the screen (‘color,’ ‘motion’) instructing subjects to attend motion and ignore color or attend color and ignore motion. Depending on the cue, after stimulus presentation (750 ms) they had to rate their degree of certainty that the net-motion of a dynamic random dot stimulus was to the left or right or that the number of dots in one color was greater than the number of dots in the other color (blue or red). After the rating subjects indicated the respective direction or color. [See **Figure 1**, Task modified from Kayser et al. (2010)]. Note that we flipped the usual order of response prompts, that is we asked for certainty-ratings first and only then let subjects indicate their binary choice. To minimize switch cost trial types were presented in Blocks of 16 trials. Block order was counterbalanced across runs and subjects. All subjects completed 5 runs of 64 trials in a pseudorandomized fashion.

### Behavioral Analysis

To analyze the effect of certainty and task on RT we specified a linear mixed model. For the analysis of effects of confidence and task on performance we used a generalized linear mixed model. In both analyses we followed an information theoretic approach via AIC comparison. To arrive at the minimum adequate model we compared a (1) full model including confidence, RT and their interaction term with 3 reduced models: (2) without interaction term, (3) only confidence term, (4) only task term.

### fMRI Data-acquisition

Whole-brain functional and anatomical images were acquired using a 3.0 T Magnetom TrioTim MRI scanner (Siemens, Erlangen, Germany) and a 12-channel head coil. A high-resolution 3D T1-weighted dataset was recorded for each participant (176 sagittal sections, 1 mm × 1 mm × 1 mm; 256 matrix × 256 matrix). Functional images were acquired using a T2^∗^- weighted, gradient-echo echo planar imaging (EPI) pulse sequence recording 37 axial slices (no gap) for whole brain coverage at an in-plane resolution of 3 mm × 3 mm × 3 mm (TE = 30 ms; TR = 2 s; FA = 70°; FoV = 192 mm × 192 mm; 64 matrix × 64 matrix). A total of 290 whole-brain volumes were recorded for each of five experimental runs of ∼10 min each.

### fMRI-preprocessing

Data quality was checked using ArtRepair^1^. Bad slices (scanner-artifacts due to Radiofrequency-coil fluctuations) were detected when the amount of data scattered outside the head (in a slice) is at least T (here default, *T* = 5) above the average amount of data scattered outside the head in the corresponding slices of the best two of the first three volumes.

Bad slices were replaced by a linear interpolation of the corresponding slices in the before and after volume. In addition data were despiked and outlier-volumes replaced by interpolating between the nearest intact volumes. For a discussion of the applied data quality check methods, please refer to Mazaika et al. (2009). We performed all analyses using MATLAB (Mathworks, Natick, MA, USA), SPM8^2^, and R^3^. fMRI data were preprocessed using standard procedures in SPM8. EPI images were realigned, coregistered to the respective participant’s T1 scan, segmented, normalized to a standard T1 template based on the Montreal Neurological Institute (MNI) reference brain, resampled to 3 mm isotropic voxels, and spatially smoothed with an isotropic 8 mm full width at half maximum (FWHM) Gaussian Kernel.

### fMRI-analysis

At the first level, we regressed fMRI time series onto a general linear model (GLM) containing stick functions representing the onset of the stimulus. Separate regressors aligned to stimulus onset modeled color and motion trials, each parametrically (linear) modulated by the reported certainty rating. Regressors were convolved with a double gamma hemodynamic response function (HRF). Motion correction parameters were entered as regressors of no interest and we applied a high-pass filter (128 s cutoff) to exclude low-frequency drifts. First-level contrast images were entered into a second-level ANOVA. The analysis included four first-level contrast images (positive and negative parametric effects of certainty in color and motion trials) from each participant. We then performed conjunction analyses (test of conjunction null hypothesis, i.e., logical AND) on the two positive and on the two negative parametric effects. All reported changes in BOLD signal survive *p* < 0.05, Family-wise-error (FWE) -corrected, at the cluster level for multiple comparisons using a cluster-defining threshold of *p* < 0.001, uncorrected.

To further specify the relation between rating-level and changes in BOLD signal, we specified an additional model. Here instead of using the rating level as a parametric modulator of one regressor, we modeled each rating level using a separate regressor. This allowed us to compute percent BOLD signal change for each rating level (e.g., **Figure 5**).

## Results

### Behavior

**FIGURE 2:**
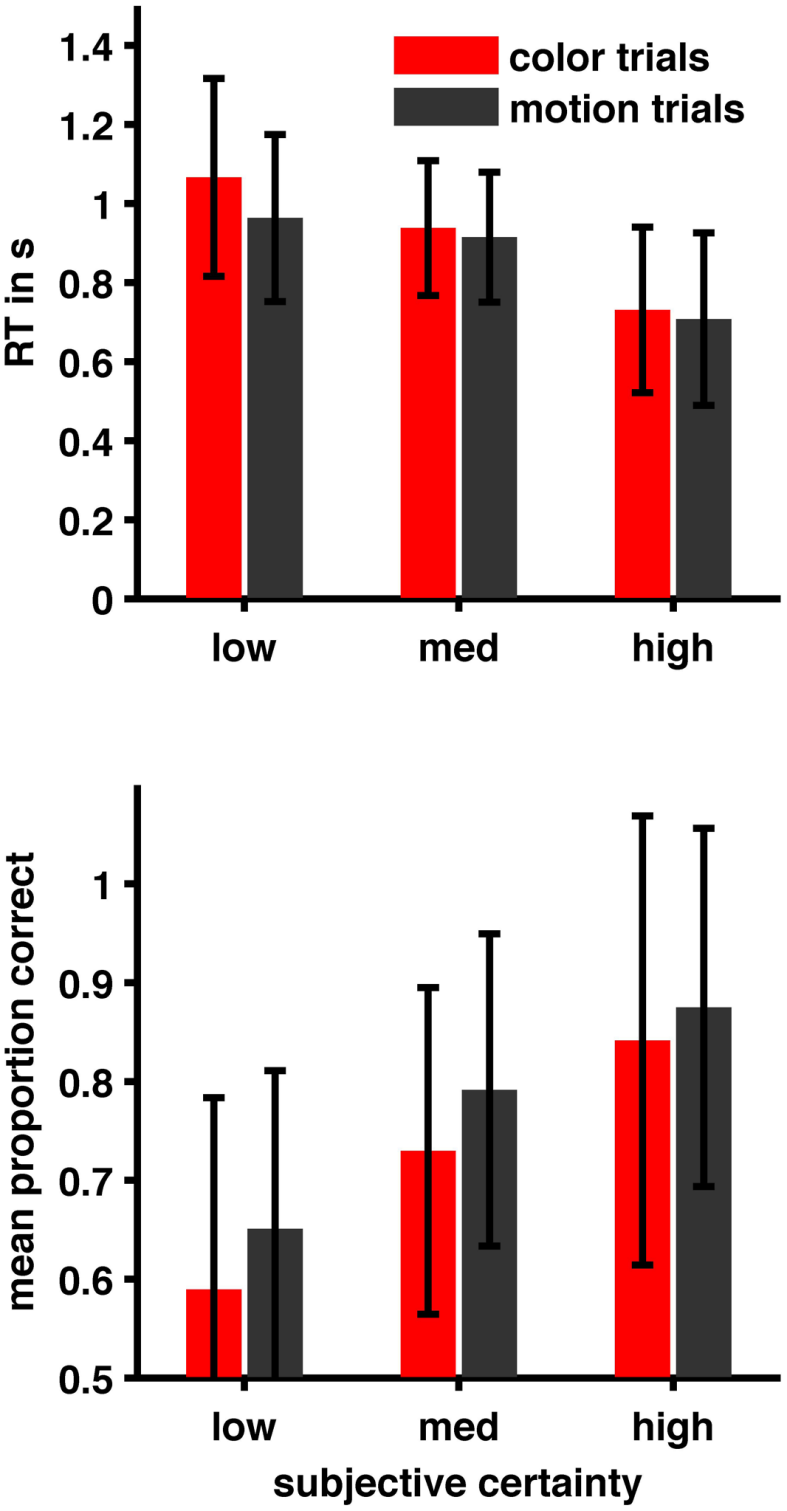
**Relation between subjective certainty ratings, performance, and reaction time**. Performance increases with subjective certainty while RT decreases (see also Results section and **Table 1**).

Subjects were correct on 80.57% (±14.99) of motion trials and 74.22% (±15.69) of color trials. Mean confidence-ratings were 2.67 (±0.25) in motion trials and 2.65 (±0.38) in color trials. Mean reaction times were 0.84 s (±0.35) in motion trials and 0.88 s (±0.36) in color trials. For the relationship between certainty, task and RT we found that a reduced model with certainty but without task as predictor and without interaction term (AIC = 7515.7) was superior to all other models. (AIC full = 7532.851, AIC task = 8495.642, AIC task + certainty = 7518.444, also see **Table 1**). For the relationship between certainty, task and performance a different reduced model (with certainty and task as predictors but without interaction term; AIC = 6512.2) was superior to all other models (AIC full = 6514.8, AIC certainty = 6551.9, AIC task = 6725.8). (See **Table 1** for details of the winning model and **Figure 1**). Given that we calibrated with respect to confidence and not to performance this latter result is to be expected. We further checked whether relationships between RT, subjective certainty and performance as expected based on the literature were present in our data. As expected, average task performance was higher for high (rating = 4 = ‘certain’) subjective certainty (color: mean = 0.8518; motion: mean = 0.885) than for low (rating = 1 = ‘uncertain’) certainty trials [color: mean = 0.5991, paired *t*-test *t*(21) = -5.777, *p* = 0.00000982; motion: mean = 0.6546, *t*(21) = -6.0991, *p* = 0.000004725] while average reaction times decreased with increasing certainty (color trials: mean *r* = -0.3357 ± 0.234 SD; motion trials: mean *r* = -0.3265 ± 0.1799 SD; **Figure 2**). Also as expected, RTs were on average longer in error-trials (color mean = 0.9272, motion mean = 0.9565) than in correct trials [color mean = 0.8723; paired *t*-test, *t*(21) = -2.2647, *p* = 0.03424, motion mean = 0.8283, paired *t*-test, *t*(21) = -4.0415, *p* = 0.0006]. Choice-RTs (time between rating and decision) were also longer in error trials (color mean = 0.5446, motion mean = 0.5309) than in correct trials [color mean = 0.4893, paired *t*-test, *t*(21) = -3.8453, *p* = 0.00094; motion mean = 0.4455, paired *t*-test, *t*(21) = -3.8423, *p* = 0.00095].

**Table 1:** Minimum adequate model for RT and performance as dependent variable.

	Log RT as dependent variable		Performance as dependent variable	
	Estimate	*SE*	Estimate	*SE*
Intercept	-0.12	0.05	0.39	0.2
Certainty 2	0.05	0.02	0.4	0.06
Certainty 3	-0.17	0.02	0.5	0.097
Certainty 4	-0.44	0.02	1.1	0.097
Task 2	-	-	1.47	0.12

*The minimum adequate model for RT has only confidence as a predictor, while the model for performance additionally includes task (color or motion) as a predictor. Intercept: mean log RT (respectively, performance) for certainty rating 1 in task 1. Certainty 2–4: increasing certainty ratings*.

In particular, we wondered whether subject-specific calibrated stimulus intensities were related to mean performance but found no significant correlation (color trials: *r* = 0.2516, *p* = 0.2587; motion trials: *r* = -0.0036, *p* = 0.9857). The same was the case for the relation between calibrated stimulus intensities and rating-RT (color trials: *r* = -0.0616, *p* = 0.7854; motion trials: *r* = 0.2767, *p* = 0.2126) as well as between intensities and subjective certainty (color trials: *r* = -0.4047, *p* = 0.0617; motion trials: *r* = -0.2720, *p* = 0.2207). We found that calibrated color and motion intensities were correlated across subjects (*r* = 0.4771, *p* = 0.0248), suggesting that we calibrated to a dimension that is shared by the color and the motion task. Also, choice-RT, i.e., the time between choice-screen onset and choice displayed a significant negative correlation with performance in color trials (*r* = -0.5053, *p* = 0.0164) but not in motion trials (*r* = -0.2910, *p* = 0.1889).

Finally, we performed a pairwise comparison of the standard deviations of the certainty-ratings in the two tasks but found no significant difference [*t*(21) = 1.885, *p* = 0.073].

### fMRI-results

Unless indicated otherwise all changes in BOLD signal are reported at a cluster-defining threshold of *p* < 0.001 (uncorrected) and family wise error (FWE) corrected for multiple comparisons at *p* < 0.05.

**Color trials:** In the color task we found a positive parametric effect of subjective certainty in the right lingual, calcarine, fusiform, and left angular gyrus. We found a negative parametric effect of certainty in the supplementary motor area (SMA) within DMPFC, superior frontal gyrus (SFG), lingual gyrus, inferior frontal gyrus (IFG), Insula, and inferior parietal lobule. (See **Table 2** for a full list of activations).

**Table 2:** Areas showing significant correlations with subjective certainty ratings in color trials (cluster-defining threshold *p* < 0.001, uncorrected; reported changes in BOLD signal corrected for multiple comparisons at *p* < 0.05, whole brain corrected).

Region	Voxels at *p* < 0.001	Peak *t*-score	*P* (cluster FWE corrected)	Peak voxel MNI coordinates
**Positive correlations**				
Lingual gyr (r)	339	5.84	<0.001	21, -70, -8
Calcarine gyr (r)		5.19		18, -82, 4
Fusiform gyr (r)		4.36		30, -67, -5
Angular gyr (l)	83	4.77	<0.019	-45, -76, 28
**Negative correlations**				
DMPFC/SMA (l/r)	1604	7.49	<0.001	-9, 11, 49
Sup front gyr (l)		6.4		-24, -4, 52
Postcentral gyr (l)		6.29		-42, -19, 52
Lingual gyr (l)	157	6.57	<0.001	-12, -88, -5
Inf occipital gyr (l)		3.9		-33, -88, -11
		3.6		-24, -91, -14
Inf front gyr (l)	260	5.43	<0.001	-45, 14, 1
		4.27		-51, 26, 28
Insula (l)		4.08		-30, 29, 4
Inf pariet lobule (r)	165	4.71	<0.001	36, -43, 52
Supramarginal gyrus (r)		3.73		33, -40, 43
Sup pariet lobule (r)		3.65		42, -31, 31
Insula (r)	82	4.43	<0.003	45, 14, -2
		4.37		33, 17, 4
Inf front gyr (r)		3.66		54, 17, 10
Precuneus (r)	69	4.24	<0.005	12, -64, 52
Middle front gyr (r)	95	3.99	<0.001	33, 38, 28
Middle front gyr (r)		3.75		27, 47, 28
Inf front gyr (r)		3.67		51, 29, 28

*FWE, family wise error; l, left; r, right; MNI, Montreal Neurological Institute; SMA, supplementary motor area; DMPFC, dorsomedial prefrontal cortex, inf, inferior; sup, superior; pariet, parietal; front, frontal; gyr, gyrus, lob, lobule*.

**Motion trials:** In the motion task we found a positive parametric effect of subjective certainty in the angular, calcarine, lingual, and fusiform gyrus, middle orbital gyrus within the ventromedial prefrontal cortex (vmPFC), and posterior cingulate cortex. We found a negative parametric effect of certainty in SMA/DMPFC, SFG, lingual gyrus, fusiform gyrus, precuneus, superior parietal lobule, inferior parietal lobule, and IFG (p. triangularis). (See **Table 3**).

**Table 3:** Areas showing significant correlations with subjective certainty ratings in motion trials (cluster-defining threshold *p* < 0.001, uncorrected; reported changes in BOLD signal corrected for multiple comparisons at *p* < 0.05, whole brain corrected).

Region	Voxels at *p* < 0.001	Peak *t*-score	*P* (cluster FWE corrected)	Peak voxel MNI coordinates
**Positive correlations**				
Angular gyr (l)	173	6.06	<0.001	-45, -70, 25
Inf pariet gyr (l)		5.3		-48, -73, 37
Calcarine gyr (r)	370	5.79	<0.001	15, -85, 1
Lingual gyr (r)		5.00		12, -76, -8
Fusiform gyr (r)		4.98		24, -73, -11
vmPFC (l/r)	562	5.74	<0.001	-3, 44, -11
		5.00		3, 35, -11
Rectal gyr (l/r)		4.57		-3, 44, -20
Posterior cingulate (l)	267	5.5	<0.001	-9, -52, 31
		4.55		-15, -52, 13
Calcarine gyr (l)		3.83		-6, -52, 7
**Negative correlations**				
SMA/DMPFC (l/r)	1970	7.09	<0.0001	-6, 8, 49
		6.04		21, 5, 67
Sup Front gyr (r)		6.01		24, 2, 58
Lingual gyr (l)	116	5.32	<0.004	-12, -88, -5
		4.69		-15, -85, -14
Fusiform gyr (l)		4.33		-24, -76, -8
Precuneus (r)	167	5.26	<0.001	9, -64, 49
		4.81		15, -76, 55
Sup pariet lob		3.73		18, -64, 61
Inf pariet lob (r)	212	4.7	<0.0001	39, -43, 46
		5.56		36, -49, 52
		4.14		33, -43, 37
Inf front gyr (l)	83	4.28	<0.019	-51, 29, 25

*vmPFC, ventromedial prefrontal cortex*.

**Conjunction null:** Testing the Conjunction null (logical AND) for the positive parametric effects we found two significant clusters. The first was centered in the right calcarine gyrus extending into the right lingual gyrus and the right fusiform gyrus. The second one was located in the left angular gyrus (see **Table 4**). Testing the Conjunction null of the certainty activation maps in both trial-types for the negative parametric effects we found four significant clusters.

**Table 4:** Conjunction: areas showing task-independent significant correlations with subjective certainty ratings in both color and motion trials.

Region	Voxels at *p* < 0.001	Peak *t*-score	*P* (cluster FWE corrected)	Peak voxel MNI coordinates
**Positive correlations**				
Calcarine gyr (r)	232	5.18	<0.001	18, -82, 4
Lingual gyr (r)		4.98		15, -76, -8
Fusiform gyr (r)		4.98		24, -73, -11
Angular gyr (l)	72	4.77	<0.032	-45, -76, 28
**Negative correlations**				
DMPFC (l/r)	1133	7.09	<0.001	-6, 8, 49
Postcentral gyr (l)		5.67		-48, -19, 55
SMA (r)		5.6		15, 11, 67
Lingual gyr (l)	78	5.32	<0.024	-12, -88, -5
Insula (r)	68	4.37	<0.04	33, 17, 4
		4.37		45, 14, -2
Inf front gyr (r)		3.66		54, 17, 10
Inf pariet lob (r)	84	4.24	<0.018	39, -43, 52
Inf pariet cortex (r)		3.48		39, -31, 37
Supramarg gyr (r)		3.41		54, -34, 46

*Cluster-defining threshold *p* < 0.001, uncorrected; reported changes in BOLD signal corrected for multiple comparisons at *p* < 0.05, whole brain corrected*.

**FIGURE 3:**
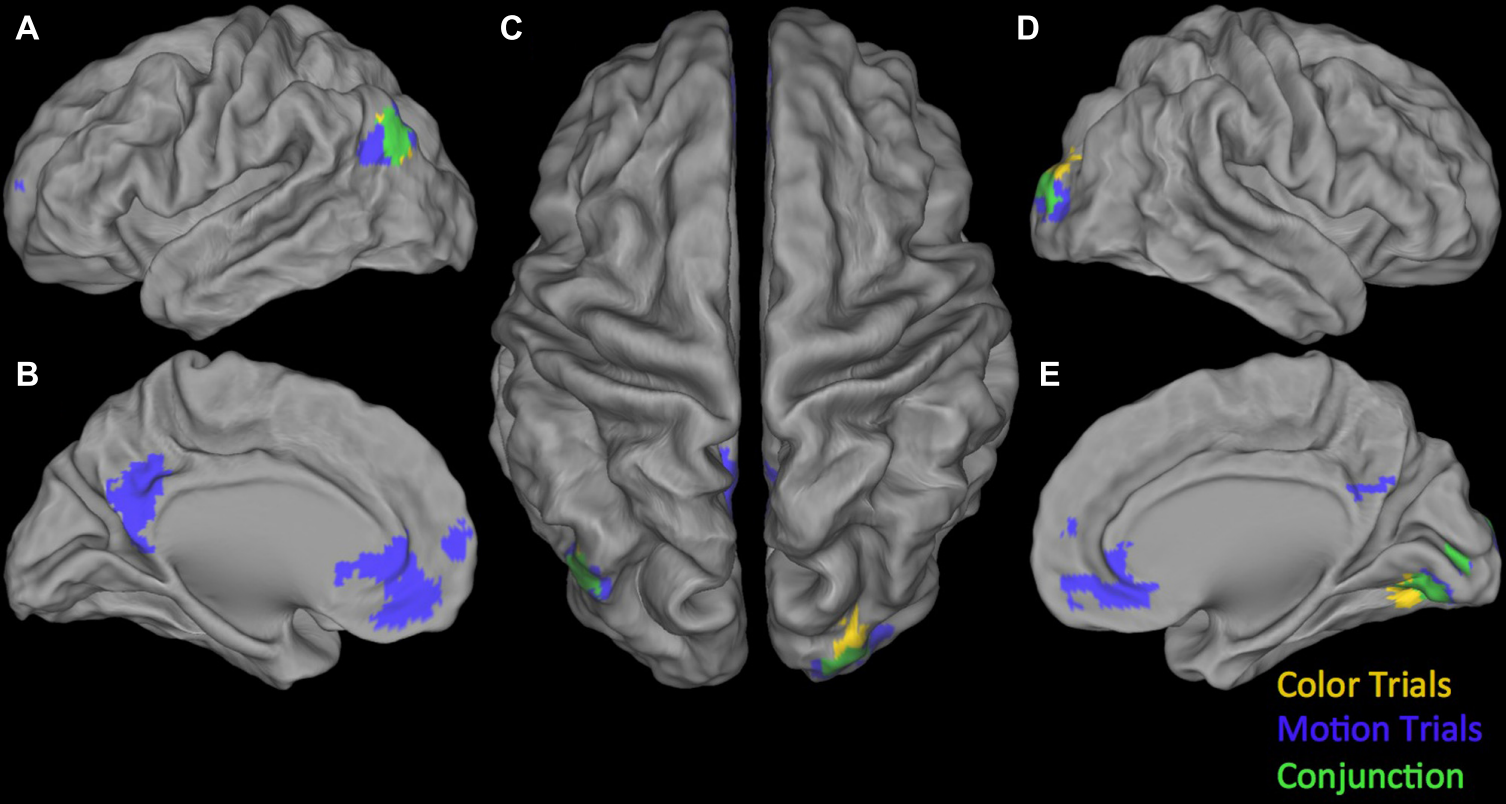
**Overview of positive parametric effects in color (yellow) and motion (blue) trials and their conjunction (green). (A)** Lateral view of left Hemisphere, **(B)** Medial view of left Hemisphere, **(C)** Dorsal view, **(D)** Lateral view of right Hemisphere, and **(E)** Medial view of right Hemisphere. See **Tables 2**–**4** for details.

**FIGURE 4:**
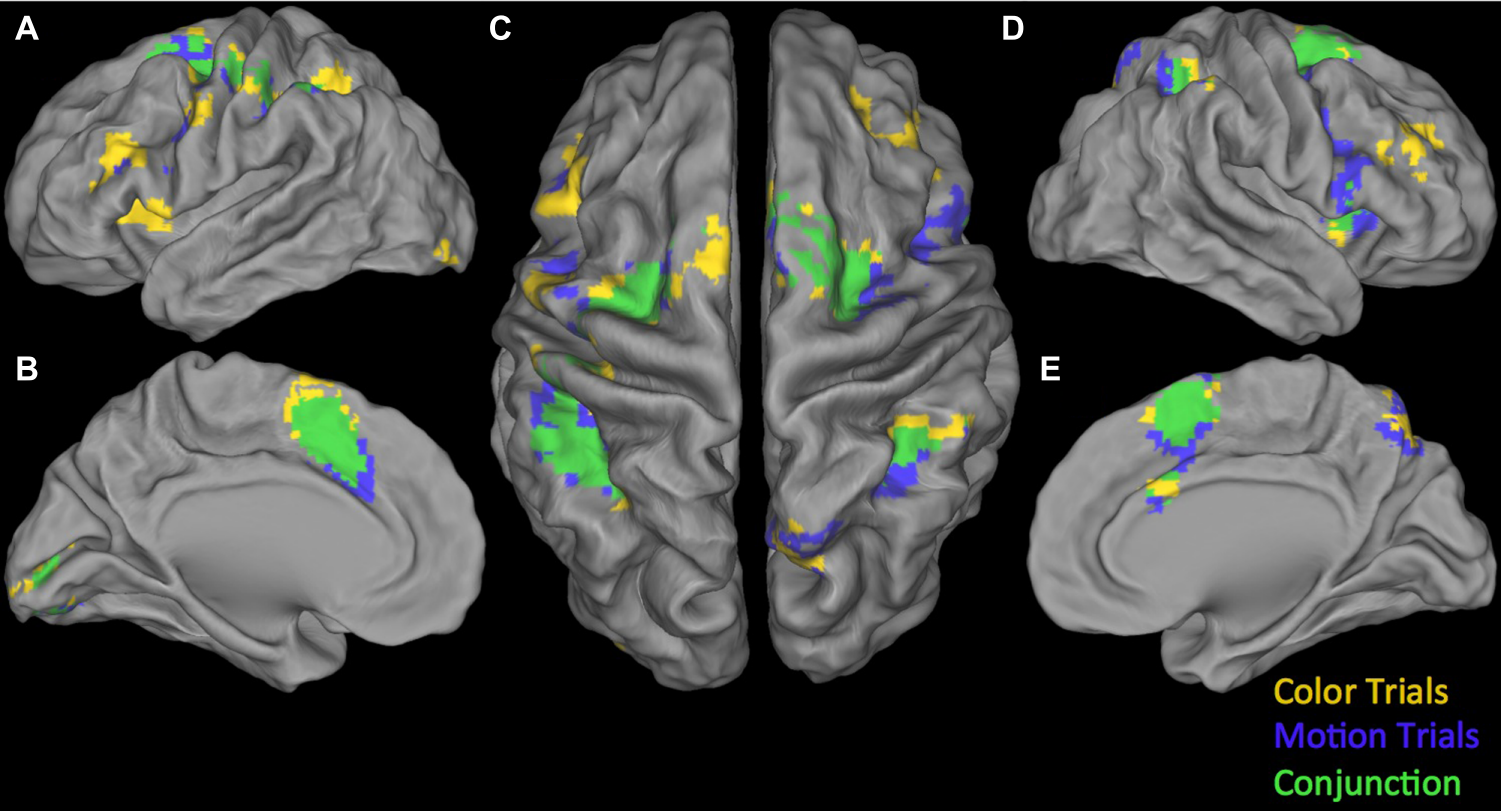
**Overview of negative parametric effects in color (yellow) and motion (blue) trials and their conjunction (green). (A)** Lateral view of left Hemisphere, **(B)** Medial view of left Hemisphere, **(C)** Dorsal view, **(D)** Lateral view of right Hemisphere, and **(E)** Medial view of right Hemisphere. See **Tables 2**–**4** for details.

The first and biggest was centered in DMPFC extending from left SMA into the left postcentral gyrus and right SMA. The others were located at left lingual gyrus, right insula, extending into right IFG (p. opercularis) and right inferior parietal lobule extending into the right supramarginal gyrus (see **Table 4**). For an overview of positive and negative parametric effects see **Figure 3** and **4** respectively.

**Control analysis:** To exclude the possibility that our main conjunction-results were biased by the rating-distribution we performed a confirmatory analysis with homogenized rating-frequencies. We randomly selected trials so that for each rating-level we used the same amount of trials. We performed this analysis within a mask generated from the contrasts from the previous parametric whole brain analysis (initial threshold *p* < 0.001, with heterogeneous rating-distributions). As not all subjects used all rating levels in all runs, for this model we did the following: If a subject in at least one run didn’t use rating level 1 (uncertain) or 2 (rather uncertain) we collapsed these two rating levels. If a subject in at least one run didn’t use rating level 3 (rather certain) or 4 (certain) we collapsed these two rating levels. We confirmed all our main results (although the exact peak-coordinates differ slightly, compare **Tables 4** and **5**) for the conjunction of the positive parametric effects (see **Table 5**).

**Table 5:** Conjunction: areas showing task-independent significant correlations with subjective certainty ratings in both color and motion trials.

Region	Voxels at *p* < 0.001	Peak *t*-score	*P* (cluster FWE corrected)	Peak voxel MNI coordinates
**Positive correlations**				
Calcarine gyr (r)	143	4.43	<0.001	15, -85, 1
Fusiform gyr (r)		4.39		24, -70, -8
Lingual gyr (r)		4.39		15, -79, -8
Angular gyr (l)	33	4.26	<0.003	-45, -76, 34
**Negative correlations**				
DMPFC/ SMA	86	5.42	<0.011	-6, 14, 49
		3.38		6, 20, 61
Calcarine gyr (l)	28	5.14	<0.017	-9, -88, -2
Lingual gyr (l)		3.73		-12, -82, -14
Postcentral gyr (l)	41	4.6	<0.007	-45, -19, 52
Sup front gyr (r)	74	4.52	<0.001	21, 5, 64
		4.51		24, 5, 52
SMA (r)		3.93		15, 11, 67
Sup front gyr (l)	26	4.52	<0.02	-21, -4, 52

*Rating frequencies are **homogenized** and the contrast is masked by the significant clusters obtained from the unhomogenized conjunction (see **Table 3**; cluster-defining threshold *p* < 0.001, uncorrected; reported changes in BOLD signal corrected for multiple comparisons at *p* < 0.05, small volume corrected)*.

**FIGURE 5:**
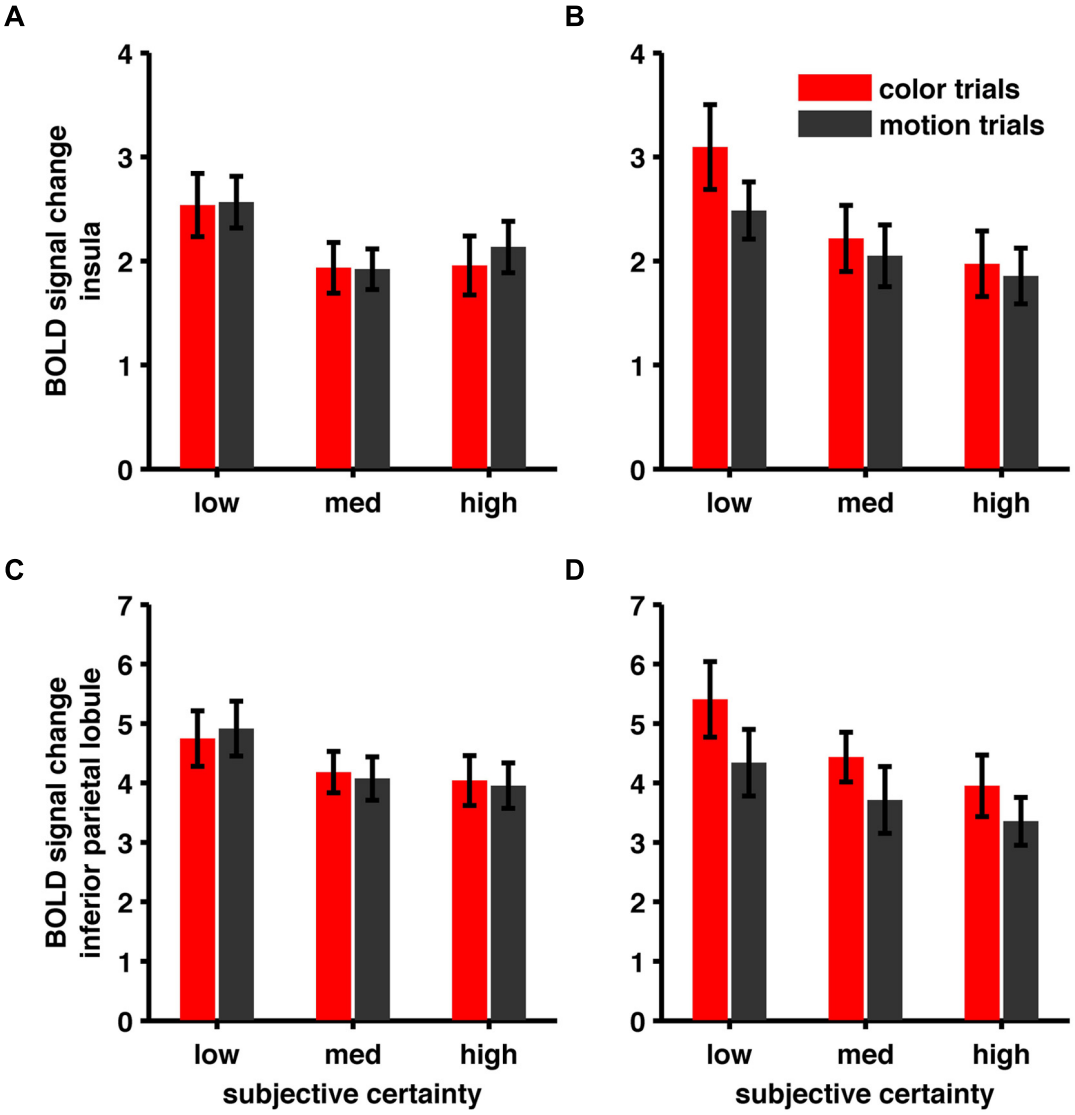
**(A)** BOLD signal change in Insula (xyz = 33, 17, 4), heterogeneous rating frequencies. **(B)** BOLD signal change in Insula, homogenized rating frequencies **(C)** BOLD signal change in inferior parietal lobule (xyz = 39, -43, 52), heterogeneous rating frequencies. **(D)** BOLD signal change in inferior parietal lobule, homogenized rating frequencies.

For the conjunction of negative parametric effects we confirmed our frontal and occipital activations, but couldn’t confirm the right insular and right parietal cluster (see **Table 5**). We wondered whether this was due to the different rating distribution or due to the reduced trial-number [and therefore a signal-to-noise-ratio (SNR) issue]. We reasoned, that if changes in BOLD signal, although non-significant, followed the same trend (i.e., higher betas for lower confidence) we would consider this an SNR-issue. For this purpose we specified an additional model, where we modeled trials associated with a specific rating level using separate regressors and extracted betas at the previously found peak coordinates in the insular cluster (xyz = 33, 17, 4) and the inferior parietal cluster (xyz = 39, -43, 52; see **Figure 5**). Both areas showed a negative parametric trend of certainty so we consider these differences in the results of the two models (heterogeneous vs. homogenized rating frequencies) to be due to SNR rather than the altered rating distribution.

In an additional control analysis we limited our analysis to correct trials only. Again, we confirmed all our main results (see **Table 6**). For further discussion we limit ourselves to changes in BOLD signal that were consistent across tasks and models.

**Table 6:** Conjunction: Areas showing task-independent significant correlations with subjective certainty ratings in both color and motion trials.

Region	Voxels at *p* < 0.001	Peak *t*-score	*P* (cluster FWE corrected)	Peak voxel MNI coordinates
**Positive correlations**				
Calcarine gyr (r)	147	5.23	<0.001	21, -82, 4
Lingual gyr (r)		4.84		15, -73, -8
Angular gyr (l)	14	3.67	<0.014	-42, -70, 37
**Negative correlations**				
DMPFC/ SMA (l)	365	5.35	<0.001	-6, 11, 49
SMA (r)		4.89		15, 11, 67
Postcentr gyr (l)	36	4.82	<0.012	-45, -19, 52
Inf pariet lob (r)	20	4.29	<0.036	36, -46, 52
lingual gyr (l)	17	4.08	<0.046	-12, -88, -5
Inf pariet lob (l)	40	3.68	<0.01	-36, -43, 46

*Here **only correct trials** are modeled and the contrast is masked by the significant clusters obtained from the ‘main’- conjunction (see **Table 3**; cluster-defining threshold *p* < 0.001, uncorrected; reported changes in BOLD signal corrected for multiple comparisons at *p* < 0.05, small volume corrected)*.

## Discussion

It is an outstanding question to what extent the neural computation of degrees of subjective certainty is task-specific. Here, we show that a substantial part of the neural representation of degrees of subjective perceptual certainty is task-invariant. We found that in two different tasks changes in BOLD signal increased with subjective certainty in the right lingual, calcarine, and left angular gyrus; BOLD signal decreased with increasing subjective certainty in the left lingual Gyrus, right inferior parietal lobule, bilateral DMPFC/SMA, and left postcentral gyrus. These changes in BOLD signal were virtually identical in the two tasks. Our data therefore support the view, that there is a central module in the brain processing subjective certainty and that, consistent with De Gardelle and Mamassian (2014), degrees of subjective certainty are represented in a task-independent format. This supports the notion of a generic neural mechanism underlying the computation of certainty.

Similarity to an deviations from other studies’ results: The observed conjunction effects in DMPFC (-6, 8, 49, MNI coordinates of peak voxel) were located closely to confidence-related changes in BOLD signal reported by Fleck et al. (2006; -11, 15, 49), Hebart et al. (2014; -9, 15, 54), and (Fleming et al., 2012; -3, 14, 46), stressing the robustness of this finding. In our motion condition we largely replicate the confidence-related findings by Hebart et al. (2014) who also used a direction-of-motion discrimination task. Importantly, however, we could not replicate these results in the color task, although we kept everything but the instruction cue constant across the two tasks. One possible explanation for this observation is that variability in certainty-ratings may be lower in the color task than in the motion task. We performed a pairwise comparison of the standard deviations of the certainty-ratings in the two tasks but found no significant difference. This suggests that differences in the activation pattern between the two tasks are genuine. Also, we did not find the positive parametric effect of certainty in the striatum reported by Schwarze et al. (2013) or Hebart et al. (2014). Regarding the role of the ventral striatum in confidence processing there are two noteworthy recent reports: Daniel and Pollmann (2012) found a positive parametric relationship between prediction error on confidence and striatal activation. Whenever confidence was higher than could be expected from previous trials, striatal activation was also higher, indicating a role of the striatum in coding changes in confidence (rather than coding confidence itself). Schwarze et al. (2013) reported that the striatum contributes to confidence processing when the task at hand is very difficult. Using an *‘unusually difficult’* task characterized by low levels of both confidence and accuracy Schwarze et al. (2013) observed a positive correlation between confidence and changes in activity in the ventral striatum. The authors explain their finding in terms of reward: Humans are typically uncertainty averse (Camerer and Weber, 1992; Hirsh et al., 2012). When difficulty is high and in consequence high-confidence trials rare, the subjective value of high confident decisions is expected to be higher than usual, i.e., subjects might experience the infrequent high confidence trials as rewarding. Combining these two findings it appears that the striatum signals when confidence is higher than expected and that this prediction error is experienced as rewarding.

As detailed in the next section, possible explanations for differences between the results of our study and those of earlier studies may be found in the details of the respective experimental designs: Relevant factors may be (a) the quantity kept constant, (b) the precise question (performance or stimulus-related) subjects are asked, (c) the time confidence is rated, and (d) whether feedback or reward were provided.

Depending on the goal of a confidence/certainty – study mainly two strategies have been used: either keep the stimulus constant or keep performance constant (e.g., using a staircase procedure). Researchers interested in subjects’ introspective ability to evaluate their performance (e.g., Lau and Passingham, 2006; Fleming et al., 2012), usually use staircase-methods to keep performance constant throughout the whole experiment (because they are interested in the relation between confidence and performance). This implies that stimulus values vary. Others, like us in the present study, are interested in the relation between stimulus and confidence, so we calibrated stimulus intensities to a predefined level of confidence (and not to a level of performance), and keep this stimulus level constant throughout the experiment. This implies that performance may vary.

Researchers interested in subjects’ introspective ability to evaluate their performance (e.g., Lau and Passingham, 2006; Fleming et al., 2012), usually use performance-related ratings (because they are interested in the relation between confidence and performance). That is: subjects rate how confident they are that their decision was correct. We asked subjects for their subjective certainty with regard to the stimulus. So we asked a different question. While the usual performance-related question (“How confident are you that your decision was correct?”) is primarily concerned with introspection and metacognition, the stimulus-related question (“How certain are you with regard to the stimulus identity?”) is of a more epistemic nature. It is concerned with the subject’s estimation of the ‘here and now’ (Komura et al., 2013) and importantly in their belief in their percept of the world.

In most recent studies subjects are asked to indicate their decision and after a forced delay indicate their degree of certainty that their decision was correct. If the primary target of investigation is choice certainty, the degree of subjective certainty associated with (i.e., directly preceding) the actual choice, this is problematic. Pleskac and Busemeyer (2010) show, that in a perceptual task evidence accumulation continues after choice, so that post-decisional confidence ratings are not based on the same evidence underlying choice. Therefore we flipped the response-order of decision and rating and asked subjects for the rating first, that is before they indicated their decision. We reasoned that already during the decision process there should be a graded certainty signal (as recently shown by Gherman and Philiastides (2015).

In contrast to animal studies most studies of confidence in humans do not give feedback or offer reward. However, some give feedback during training (e.g., Hebart et al., 2014) which may still affect neural processes in the main experiment. Varying these four factors (quantity kept constant, performance- vs. stimulus-related question, time of rating, and feedback/reward) in a systematic and independent fashion could shed light on the origins of the observed differences in experimental results.

One possible shortcoming of this study is that variations in certainty might partially be due to fluctuations in attention. However, it has been shown (Macdonald et al., 2011) that attention and confidence, although they are both related to performance, are not necessarily correlated. In addition, although we held stimulus information constant, there are fluctuations in the momentary evidence, which may partially explain the observed variation in subjective certainty at an otherwise fixed stimulus-level [For effects of the temporal distribution of evidence on confidence see Zylberberg et al. (2012)]. However, this doesn’t affect that mean-coherence, and therefore average information available to the subject, was constant.

In the present study we asked subjects for their perceptual certainty before choice and we observed a parametric modulation of BOLD signal by certainty-ratings already during stimulus presentation. Using EEG, Gherman and Philiastides (2015) showed that a confidence-signal, which could not be explained by stimulus difficulty or performance, emerges as early as the decision process itself. Similar results were obtained by Zizlsperger et al. (2014) showing that a perceptual confidence-signal, which is dissociable from representations of sensory evidence and performance, is present as early as 300 ms after stimulus onset. The temporal aspect of these results clearly challenges the generality of the metacognitive account of confidence/certainty. According to this account (e.g., Nelson, 1990; Fleming et al., 2010), confidence is modeled as the result of a noisy read-out of a decision variable. This model therefore excludes the existence of ‘pre-decision-confidence’ as observed by us and Gherman and Philiastides (2015). The existing literature makes it unlikely that confidence is a purely post-decisional process. Its processing might either start pre-decisionally or alternatively, pre- and post- decisional certainty processing may serve different purposes and may be based on partially different mechanisms. While pre-decision certainty is associated with incoming evidence and the actual decision process, post-decision confidence may be best viewed as an error-detection-signal (Boldt and Yeung, 2015) and, as suggested by Hebart et al. (2014), provide a learning signal in the absence of feedback. Whether this signal is metacognitive in nature and the result of a noisy read-out of the decision variable at the time of choice is unclear. Yu et al. (2015) report compelling evidence for post-decisional processing of confidence. Specifically, they show that with longer forced delays between decision and rating the resolution of the confidence-accuracy relationship increases. While confidence in correct responses stays relatively stable over different forced-delay durations, confidence in incorrect responses decreases with increasing forced delay durations. This is in line with research on the link between decision confidence and error detection (Macdonald et al., 2011; Boldt and Yeung, 2015) and at the same time challenges the metacognitive account of confidence. Further research and a formal comparison of process models are needed to shed light on this issue.

In the present study even when in addition to the stimulus, performance was held constant, we still observe parametric effects of certainty (see **Table 6**). This indicates that the processes we observe here are not directly performance-related. The observation of stimulus- and performance-independent certainty signals in our study and reported above (Zizlsperger et al., 2014; Gherman and Philiastides, 2015) is further supported by McSorley et al. (2014). They report eye-tracking-evidence for the claim that in perceptual decision making, decision and confidence are based on different information sources or processing mechanisms. In line with this dissociability, Vlassova et al. (2014) show that unconscious information changes decision accuracy but not confidence. The dissociability of performance- and confidence-related processes gets further support from the animal literature: Komura et al. (2013) report that silencing of the pulvinar nucleus decreased monkeys’ confidence without affecting performance. Similarly, Lak et al. (2014) found that orbitofrontal cortex inactivation disrupts confidence processing without affecting decision accuracy. However, Fetsch et al. (2014) report opposing results suggesting that the same neural signals support choice, reaction time, and confidence in a decision. Taken together the aforementioned findings indicate that behavioral confidence and performance do not necessarily go hand in hand. On the neural level the picture is more complex: Komura et al. (2013), Fetsch et al. (2014), and Lak et al. (2014) recorded or manipulated neural activity at different sites (LIP, pulvinar thalamic nucleus, and OFC). Taken together, their results suggest that within the brain network processing confidence and performance, some nodes may represent confidence and performance jointly (e.g., LIP), while other nodes represent these quantities separately (e.g., pulvinar and OFC). This view accommodates the observations, that on the one hand confidence and performance are usually correlated, and that on the other hand there are instances in which they are not.

**FIGURE 6:**
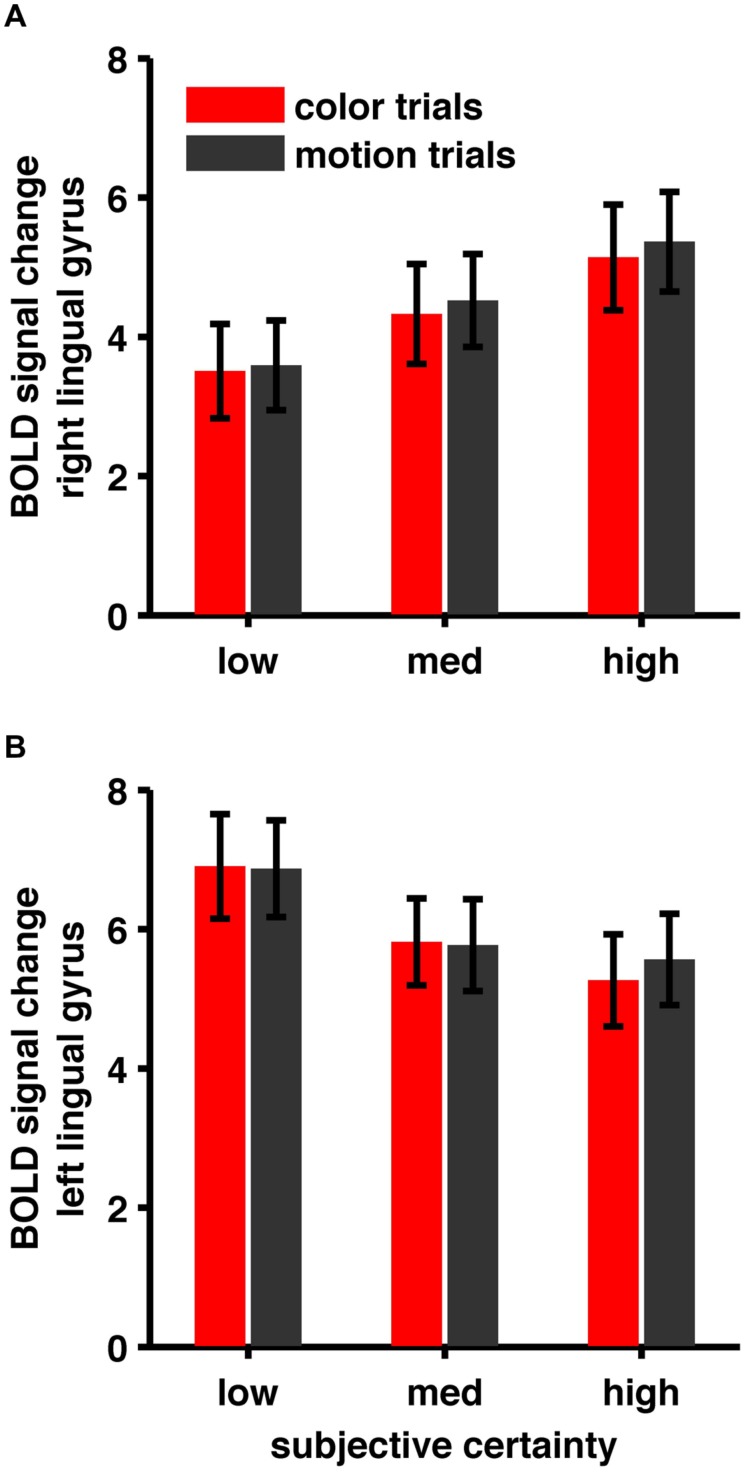
**(A)** BOLD signal change in right lingual gyrus, **(B)** BOLD signal change in left lingual gyrus. Areas show opposite patterns. While BOLD signal in right lingual gyrus shows a positive correlation, BOLD signal in left lingual gyrus shows a negative correlation with subjective certainty ratings.

One particularly interesting observation is that we find both, areas showing a positive and areas showing a negative task-independent parametric effect of subjective certainty (see **Figure 6** for an example). While some authors only report negative parametric effects (e.g., Fleming et al., 2012), others report both (Hebart et al., 2014) but focus on regions displaying a positive parametric effect of confidence. The usual claim is, that either areas showing a positive effect or areas showing a negative effect represent confidence proper.

In our view the co-observation of these effects raises an intriguing alternative explanation: Imagine the brain is in a very high (or very low) activity state. A simple one-directional mechanism where certainty is computed in a way that increasing certainty is associated with increasing neural activity, would loose its calibration or better, its capability to adequately code certainty. If alternatively, certainty (as observed here) is computed in an interplay of increases and decreases in neural activity, the baseline state of brain activity is largely canceled out and the mechanism preserves its calibration. Also, a mechanism where certainty computation is realized by one principle alone is vulnerable and therefore prone to error. In contrast, a mechanism where certainty is computed in parallel (i.e., redundantly) and in several different ways would be very robust against system perturbation.

Finally, our results not only shed light on the neural representation of subjective certainty but by showing evidence for task-independence of certainty-processing also legitimize a broader and more general interpretation of previous studies of certainty. However, further research is needed to clarify the generalizability of our findings to auditory and somatosensory settings, as well as to non-perceptual tasks.

## Conflict of Interest Statement

The authors declare that the research was conducted in the absence of any commercial or financial relationships that could be construed as a potential conflict of interest.

## Footnotes

1 http://cibsr.stanford.edu/tools/human-brain-project/artrepair-software.html.

2 http://www.fil.ion.ucl.ac.uk/spm/

3 https://www.r-project.org/.
